# Autophosphorylation at Thr279 of *Entamoeba histolytica* atypical kinase EhAK1 is required for activity and regulation of erythrophagocytosis

**DOI:** 10.1038/srep16969

**Published:** 2016-01-07

**Authors:** M Shahid Mansuri, Mrigya Babuta, Mohammad Sabir Ali, Ravi Bharadwaj, Gagan Deep jhingan, Samudrala Gourinath, Sudha Bhattacharya, Alok Bhattacharya

**Affiliations:** 1 School of Life Sciences, Jawaharlal Nehru University, New Delhi 110067, India; 2 School of Biotechnology, Jawaharlal Nehru University, New Delhi 110067, India; 3 National Institute of Immunology, New Delhi 110067, India; 4 School of Environmental Sciences, Jawaharlal Nehru University, New Delhi 110067, India

## Abstract

Phagocytosis plays a key role in survival and pathogenicity of *Entamoeba histolytica*. We have recently demonstrated that an atypical kinase EhAK1 is involved in phagocytosis in this parasite. It is recruited to the phagocytic cups through interaction with EhCaBP1. EhAK1 manipulates actin dynamics by multiple mechanisms including phosphorylation of G-actin. Biochemical analysis showed that EhAK1 is a serine/threonine kinase with broad ion specificity and undergoes multiple trans-autophosphorylation. Three autophosphorylation sites were identified by mass spectrometry. Out of these Thr279 appears to be involved in both autophosphorylation as well as substrate phosphorylation. Over expression of the mutant Thr279A inhibited erythrophagocytosis showing dominant negative phenotype. Multiple alignments of different kinases including alpha kinases displayed conserved binding sites that are thought to be important for function of the protein. Mutation studies demonstrated the importance of some of these binding sites in kinase activity. Binding studies with fluorescent-ATP analogs supported our prediction regarding ATP binding site based on sequence alignment. In conclusion, EhAK1 has multiple regulatory features and enrichment of EhAK1 at the site of phagocytosis stimulates trans-autophosphorylation reaction that increases kinase activity resulting in enhanced actin dynamics and phagocytosis. Some of the properties of EhAK1 are similar to that seen in alpha kinases.

Detailed molecular mechanism of phagocytosis by the protist parasite *Entamoeba histolytica* is not yet clear though a few of the molecules that participate in the process has been identified^1^,^2^,^3^,^4^. However, a number of molecules, particularly those that are involved in different signalling pathways leading to phagosome formation are still unknown. We have identified a few molecules that are involved in *E. histolytica* phagocytosis and investigated the mechanisms by which these molecules participate in phagocytic process using uptake of red blood cells (RBC) as a system^5^,^6^,^7^. Our results suggest a novel pathway involving calcium binding proteins EhCaBP1 and EhCaBP3 along with protein kinases EhC2PK and EhAK1. Since down regulation of expression of any one of these proteins can block phagocytosis and consequently cell proliferation, detailed molecular characterization of the phagocytic pathway may be of use in identifying novel targets for development of new therapeutics.

EhAK1 was identified as one of the EhCaBP1 binding proteins by affinity chromatography and mass spectrometry^6^. EhAK1 has two main domains, an alpha kinase like kinase domain and a SH3 domain. A number of experiments showed that it indeed binds EhCaBP1 in presence of Ca^2+^ and the binding is through the kinase domain. However, the binding does not require active kinase domain. Therefore the kinase dead mutant can also bind EhCaBP1. However, progression of phagocytic cups to phagosomes require active kinase domain as over expression of inactive kinase domain of EhAK1 displayed dominant negative phenotype with respect to phagocytosis^8^. Moreover, EhAK1 phosphorylates actin at threonine 107 and it is an important step in progression of phagocytic cups to phagosomes. Our data had suggested that this phosphorylation alters dynamic properties of actin, thereby helping phagocytic process^8^. Since EhAK1 displays novel domain organization and similar kinases have not been reported to be involved in phagocytosis, we have investigated biochemical properties of EhAK1 in order to understand molecular basis of some of the atypical properties of this amoebic kinase in relation to its role in *E. histolytica* phagocytosis.

Our results show that EhAK1 displays multiple trans-autophosphorylation needed for optimal and unique activity of the kinase. Different autophosphorylation sites appear to have different roles in EhAK1 activity. Our data suggest that phosphorylation of Thr279 is likely to have a major role in the function of EhAK1.

## Results

### Phosphorylation activity of EhAK1

**Figure 1:**
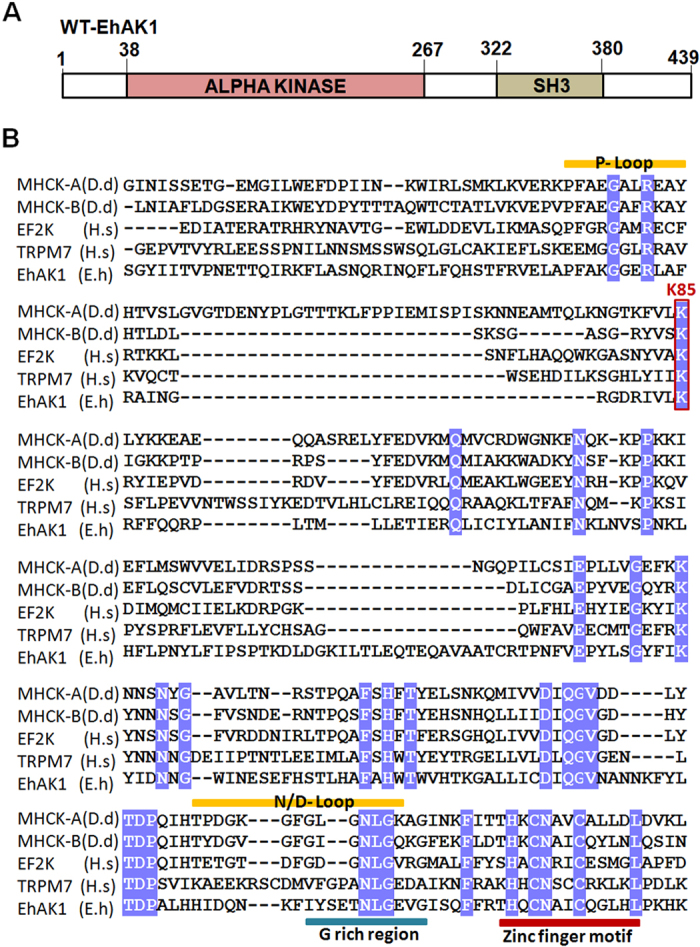
Domain organization and multiple sequence alignment of the kinase domain of EhAK1. (**A**) Schematic representation of domain organization of EhAK1. EhAK1 is a 50 kDa protein with two domains, alpha kinase domain (38–267 amino acids) and SH3 domain (322–380 amino acids). (**B**) Alignment of the sequences of kinase domains of EhAK1, MHCK-A, MHCK-B, EF2 K, and TRPM7. The kinase domains of EhAK1, MHCK-A, MHCK-B, EF2 K, and TRPM7 (UniprotKB accession codes C4M9G9, P42527, P90648, 000918 and Q923J1, respectively) were aligned on the basis of similarities in their sequence. Sequences representing P-loop, N/D loop, G-rich region and Zinc finger motif are indicated. Invariant K85 that is predicted to be involved in nucleoside binding site is also shown. Identical residues in EhAK1 are shown in white text on a blue background.

**Figure 2:**
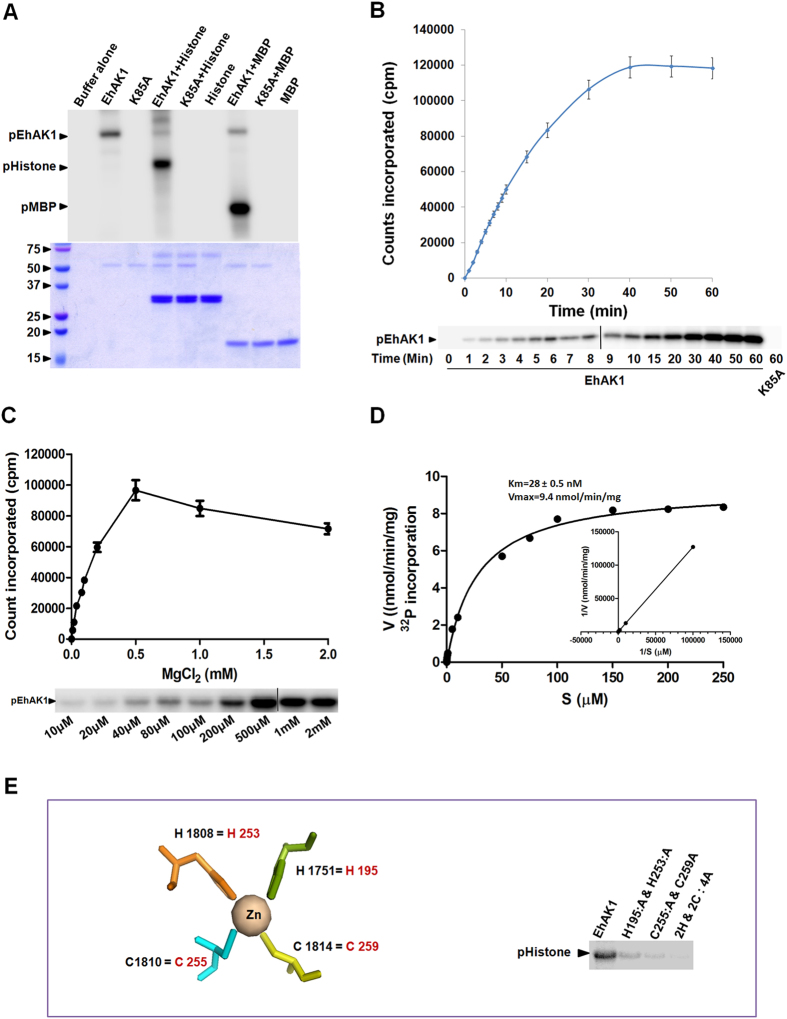
Phosphorylation activity of EhAK1. (**A**) Autophosphorylation and substrate phosphorylation activities of His-EhAK1. Each reaction was set with 2 μg of recombinant tagged EhAK1 or K85A in the presence of γ-^32^P-ATP, MgCl_2_ at 30 °C for 1 h in kinase assay buffer and for substrate 2 μg of histone type (IIIS) or myelin basic protein (MBP) was used. K85A mutant of EhAK1 was used as a control. The kinase reaction was stopped by adding Laemmli buffer with 1 mM EDTA. The products were analysed on SDS-PAGE and visualized using a phosphorimager or gel was stained by coomassie brilliant blue and bands were cut and counts were taken in a scintillation counter. (**B**) Time kinetics of kinase activity. The autophosphorylation activity of His tagged EhAK1 was carried out as described in panel 1A and reaction was incubated for indicated period of time in kinase assay buffer. (**C**) Optimisation of Mg^2+^ concentration. The autophosphorylation activity of His tagged EhAK1 was carried out as described in panel 1A, in presence of indicated concentration of MgCl_2_. (**D**) Michelis-Menten parameters for wild-type EhAK1. Vmax for ATP was determined to be 9.4 nmol/min/mg protein and Km was 28 nM. The cold ATP was varied from 0.4 pmol to 8 μmol in presence of 10 μCi [^32^P-γ]ATP. The protein was phosphorylated for 1 h at 30 °C. The amount of radioactivity incorporated was determined. All the points were in duplicates. The experiment was repeated three times. (**E**) Role of zinc binding residues in activity of EhAK1: Upper panel shows the schematic representation of Zn^2+^ and coordination with residues (Black) in TRPM7 (PDB-1IAJ). Conserved residues present in EhAK1 are shown in red. Lower panel shows the histone phosphorylation activity describe in panel 1A, of wild type, H195A , H253A, C255, C259A and all four mutants (2H & 2C: 4A) of EhAK1 were checked.

EhAK1 does not display extensive sequence similarities with other kinases like typical protein kinases. However, most of the key functional residues are generally conserved and this conservation of key functional residues has also been seen in EhAK1 when compared with a few distant homologs based on sequence conservation (Fig. 1A,B). Kinase activity was tested *in vitro* using histone type (IIIS) and myelin basic protein (MBP) as substrates. Phosphorylation was observed with both wild type full length protein (His-EhAK1) and the kinase domain (GST-KD) but not with the mutant protein (K85A) (Fig. 2A and S1). K85 has been predicted to be one of the key residues involved in nucleotide triphosphate binding^8^,^9^. Involvement of this residue in NTP binding has also been shown experimentally (see letter section). The time kinetics of autophosphorylation of the full-length protein showed an increase in the rate of phosphorylation leading to saturation at about 40 min (Fig. 2B). Therefore, all kinase reactions were carried out for 1 h. Furthermore, we immunoprecipitated autophosphorylated EhAK1 by different phospho-amino acid-specific antibodies [anti phospho-serine (Ser)/threonine (Thr)/tyrosine (Tyr) antibodies] in order to determine specificity of phosphorylation reaction carried out by EhAK1. The phosphorylated bands were recognized by anti p-Ser and anti p-Thr antibodies, but not by anti p-Tyr antibodies (Fig. S2). No band was visible in the lane where control serum was used (Fig. S2). These results suggest that EhAK1 is a Ser/Thr kinase.

Both full-length protein and the kinase domain displayed autophosphorylation along with substrate phosphorylation (Fig. 1A and S1). We also checked for divalent cation requirement in the phosphorylation reaction. Auto-phosphorylation as well as substrate phosphorylation activities of EhAK1 were maximum in the presence of Mg^2+^ ions (0.5 mM MgCl_2_) (Fig. 2C, S3A and S3B). Significant activity was also observed in presence of other divalent ions, such as Mn^2+^, Co^2+^, Zn^2+^ and Ni^2+^(Fig. S3A and S3B). Km and Vmax for ATP were estimated to be 28.0 nM and 9.4 nmol/min/mg ATP, respectively (Fig. 2D). These values (particularly Km) are similar to that seen for Ser/Thr protein kinases (10–150 nM)^10^,^11^. Moreover, we checked the effect of genistein (tyrosine kinase inhibitor) and staurosporine (serine/threonine kinase inhibitor) on EhAK1 activity. There was no significant inhibition of autophosphorylation by these inhibitors at nontoxic concentrations (Fig. S4A and S4B) confirming atypical nature of this kinase.

EhAK1 carries other conserved sites based on multiple alignments shown in Fig. 1B (Zinc finger motif, P-loop, N/D region and G-region). Importance of this motif (amino acids 252 to 264) was demonstrated by generating mutations (double and tetra) in the residues H195A & H253A, C255A & C259A, 2H & 2C: 4A. These mutations resulted in a reduction of the kinase activity by 75%, 80% and 85% respectively, compared to the wild type EhAK1 (Fig. 2E). The actual role played by the putative zinc finger motif in regulating kinase reaction is not immediately apparent but our data suggest that this motif is required for kinase activity of EhAK1. More ever, this site was also found to be conserved in alpha kinases and it is thought to bind Zn^2+^ and has kinase regulatory role^9^.

### Analysis of ATP binding sites of EhAK1

A probable ATP binding site in EhAk1 was deduced from sequence alignment (Fig. 1B)^8^. The site was found to be conserved when sequences from a number of kinases were aligned. This was further investigated *in vitro* by fluorescence spectroscopy using TNP-ATP, an analog of ATP. The three conserved key residues Lys 85, Asp 223 and Arg 69 needed for kinase activity were mutated to alanine by site-directed mutagenesis. Tryptophan fluorescence was used for monitoring conformational changes in EhAK1. ATP binding reduced tryptophan fluorescence of the protein similar to that seen for EhC2PK^6^. With increasing amounts of ATP, fluorescence decreased by 76, 74, 30, and 16% of the wild type EhAK1, D223A, R69A, and K85A mutants, respectively (Fig. S5).

**Figure 3:**
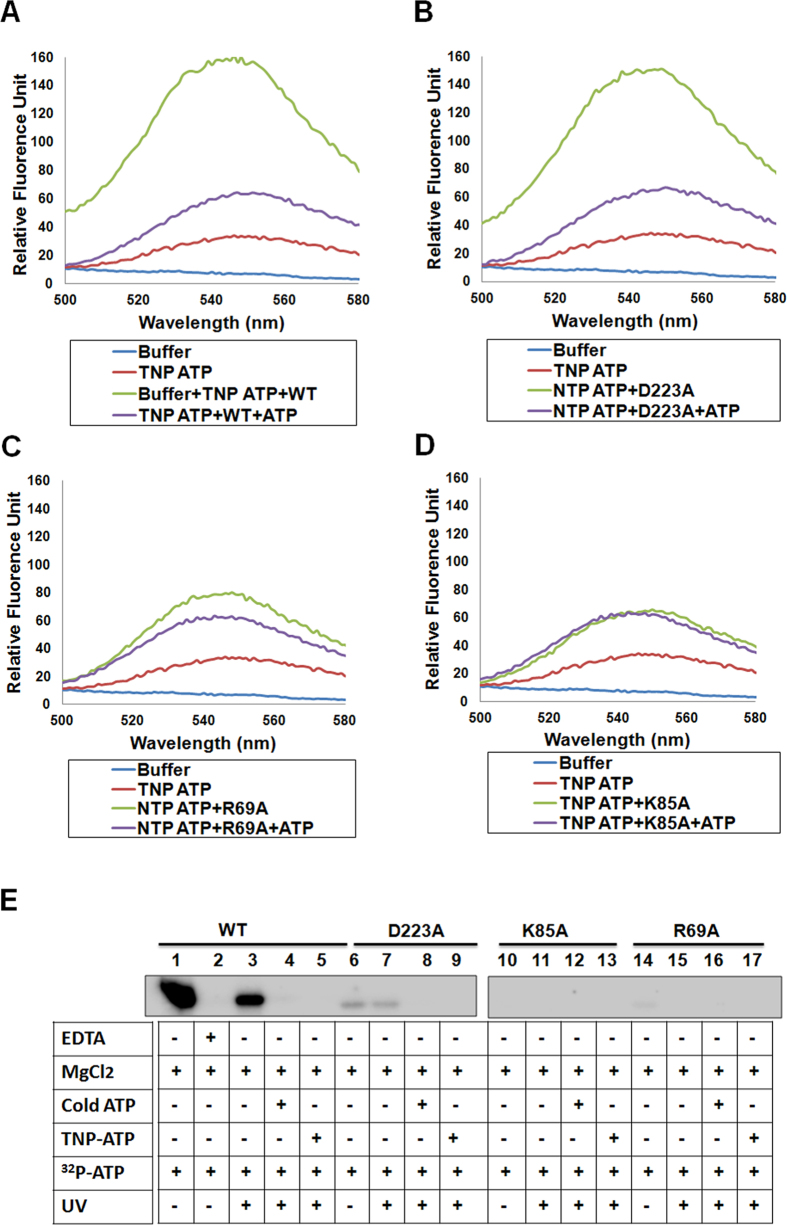
Interaction of ATP with EhAK1. (**A–D**) Fluorescence emission spectra of TNP-ATP (10 μM) in 5 mM HEPES buffer containing 1 mM MgCl2 and 100 mM NaCl with (green) or without (red) wild type EhAK1 (2.5 μM) (B), D223A (**C**), R69A (**D**) and K85A (**E**). Subsequent addition of 5 mM ATP (blue) causes shift of the graph close to TNP-ATP alone. ATP binding by wild type EhAK1, D223A mutant, R69A mutant and K85A mutant was checked by UV cross linking method. Wild type EhAK1, D223A mutant and K85A mutants were incubated with ^32^P-ATP and cross linked with UV light. Lane 1 has autophosphorylated protein while in lane 2 kinase reaction is inhibited by EDTA. Also the same is competed by cold ATP in lane 4 and TNP-ATP in lane 5. Lane 6, lane 10 and lane 14 display autophosphorylation reaction with D223A, K85A and R69A mutants which are not active. Lane 7 shows reduced ability of mutants for ATP binding as compared to wild type and was absent in lane 11 and 15. Also ^32^P-ATP cross linking to mutants is competed out by cold ATP in lanes 8, 12 and 16 and by TNP ATP in lanes 9, 13 and 17.

ATP binding was also monitored by using fluorescent ATP analogue TNP-ATP (2′, 3′-O-(2,4,6 trinitrophenyl)-adenosine triphosphate) by competition assay. TNP-ATP displays high degree of stimulation in fluorescence emission upon binding to proteins as compared to only buffer; absolute magnitude is dependent on the specific protein environment^12^. For the wild-type EhAK1 and the D223A mutant, there was a 5-fold increase in fluorescence of TNP-ATP in presence of respective proteins (Fig. 3A,B). This increase was not seen in the presence of 500-fold excess ATP. Fluorescence did not change significantly when R69 and K85 mutants were incubated with TNP-ATP (Fig. 3C,D). These results suggest that both K85 and R69 residues are involved in ATP binding.

This was further confirmed by UV cross-linking of γ-^32^P-ATP with purified proteins. The wild type, K85A, D223A and R69A were incubated with γ-^32^P-ATP and irradiated with UV (254 nm) in order to cross-link proteins with radiolabeled ATP. While cross-linking of γ-^32^P-ATP with the wild type and D273A mutant was observed (radioactive band in autoradiogram), only low level of cross-linking was visible in the case of R69 and none when K85 mutant was used (Fig. 3E). This study supports that K85A and R69A mutants are impaired in ATP binding. The affinities of ATP towards K85A and R69A mutants were found to be much lower than that of the wild-type protein.

### Identification of autophosphorylation sites of EhAK1

**Figure 4:**
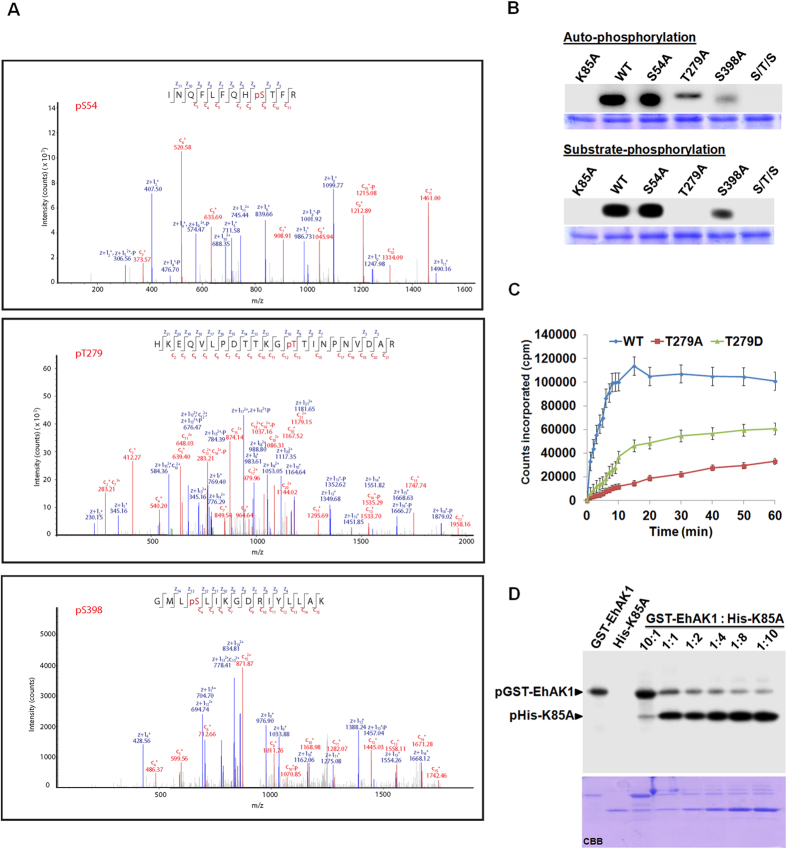
Identification of autophosphorylation sites of EhAK1. (**A**) LC-MS/MS data showing Electron transfer dissociation (ETD) induced fragmentation mass spectra identifying three phosphopeptides of EhAK1. *pT* indicates the site of phosphorylation. MS/MS spectrum of precursor *m/z: 539.92596* (+3) and MH+: 1617.76334 Da, of phosphopeptide INQFLFQH(pS)TFR. Unambiguous location of the intact phosphate group on Ser-54 was evident by the observation of the “C” ion series (C9-C11)and the “Z” ion series (Z4-Z11); pRS score 239, pRS probability 100%. MS/MS spectrum of precursor *m/z: 629.31378* (+4) and MH+: 2514.23330 Da, of semi-tryptic phosphopeptide HKEQVLPDTTKG(pT)TINPNVDAR. Unambiguous location of the intact phosphate group on Thr-279 was evident by the observation of the “C” ion series (C13-C21) and the “Z” ion series (Z10-Z21),pRS score 142, pRS probability 98.3%. MS/MS spectrum of precursor *m/z: 624.34515* (+3) and MH+: 1871.02091 Da, of semi-tryptic phosphopeptide GML(pS)LIKGDRIYLLAK. Unambiguous location of the intact phosphate group on Ser-398 was evident by the observation of the “C” ion series (C4-C15) and the “Z” ion series (Z13-Z14),pRS score 171, pRS probability 100% (**B**) Autophosphorylation and Substrate phosphorylation of recombinant protein. The identified autophosphorylation sites were mutated by site directed mutagenesis to alanine. The autophosphorylation was carried out using 2 μg of recombinant proteins. Substrate (histone) phosphorylation was determined using 2 μg of indicated proteins in presence of 5 μg of histone. (**C**) Time course for substrate (5 μg) phosphorylation activity of 2 μg of purified wild type (blue line), T279A (red line), and T279D (green line) of EhAK1. (**D**) Autophosphorylation of EhAK1 by cross phosphorylation reaction. Purified GST-EhAK1 (donor) and His-K85A (accepter) were used for the assay. Lane 1: shows autophosphorylation activity of wild type GST-EhAK1. Lane 2: His-K85A mutant as control. Lane 3: phosphorylation of His-K85A mutant by wild type GST-EhAK1. K85A has intact phosphorylation site but it is a kinase dead mutant. Lane 4–8: phosphorylation of K85A by wild type GST-EhAK1 with increasing amount of K85A at fixed concentration of wild type GST-EhAK1. The kinase reaction was stopped by adding Laemmli buffer with 1 mM EDTA. The products were analysed on SDS-PAGE and visualized in a phosphorimager.

Autophosphorylation sites were identified by mass spectrometry of trypsin digested purified EhAK1, incubated with ATP in phosphorylation buffer without any external substrate. The results revealed mainly three phosphorylated peptides. Sequencing of the peptide fragments identified Ser 54, Ser 398 and Thr 279 as the major phosphorylated amino acids suggesting that these residues are likely to be autophosphorylation sites (Fig. 4A and Table S1). A detailed study of the spectra also revealed the presence of the same peptide(s) in a non-phosphorylated form. The mass of these peptides were less by 80 daltons as compared to that of phosphorylated ones. We have concentrated on these three sites for further experiments.

Mass spectrometric prediction of phosphorylation sites was confirmed by generating point [S54A, T279A, S398A and S/T/S-(3A)] mutants. The purified mutant proteins were tested for autophosphorylation, and the results are shown in Fig. 4B. The mutant proteins exhibited significant reduction in total autophosphorylation activity (30% for S54A, 70% in T279A and 80% for S398A) as compared with the wild-type protein (Fig. 4B). Since total combined inhibition of autophosphorylation (S/T/S-3A triple mutant) was about 100% it appears that there is some interaction among different sites, that is, mutation in one can affect autophosphorylation of another site (Fig. 4B). The mutants were also tested for their ability to phosphorylate substrates. While there was no significant change in activity of S54A (5%) and a reduction in activity of S398A (50%) with respect to the wild type protein, T279A did not phosphorylate the substrate at all (Fig. 4B). The results suggested that autophosphorylation at Thr279 is required for substrate phosphorylation by EhAK1. We also generated putative phosphomimetic mutant T279D and used it in kinase reactions. Time-kinetics of substrate phosphorylation by T279D displayed a slow linear initial rate of substrate phosphorylation and lack of saturation phase unlike the wild-type protein (Fig. 4C). After the slow initial phase there was a linear increase in product formed during the time frame used for this study (Fig. 4C). These results suggest that autophosphorylation at Thr 279 is an important regulator of EhAK1 activity.

### Trans-reaction mediates EhAK1 autophosphorylation

Autophosphorylation of the kinase domain can be either through a unimolecular cis-reaction or a bimolecular trans-reaction. To check this, phosphorylation was carried out with GST-tagged EhAK1 (donor) and His-tagged kinase dead mutant K85A (acceptor) (Fig. 4D). Since only the His-tagged protein was phosphorylated it appears that auto-phosphorylation takes place by trans-bimolecular reaction. This was further demonstrated by using varying amounts of donor (GST-EhAK1) and acceptor (K85A) mutants (Fig. 4D). On increasing the acceptor molecules in relation to a constant level of donor molecules, the autophosphorylation of the acceptor increased (Fig. 4D). A small amount of autophosphorylation, observed in the donor molecule, was significantly reduced as the concentration of acceptor molecules was increased (Fig. 4D). These results suggest that EhAK1 autophosphorylates using a bimolecular trans-reaction.

### Phosphorylation at Thr279 is essential for phagocytosis

**Figure 5:**
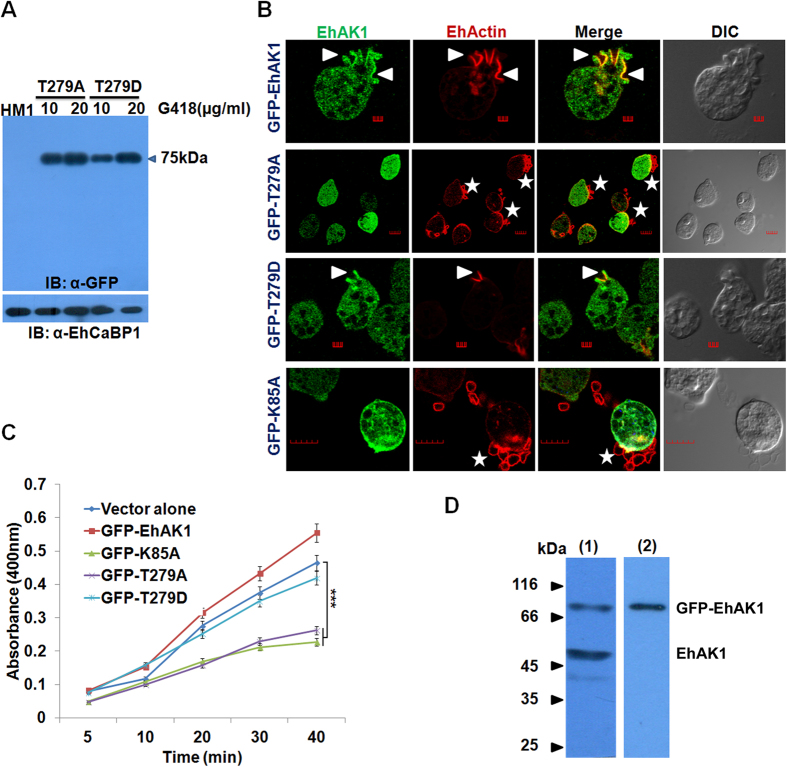
Role of phosphorylation at T279 of EhAK1 during phagocytosis. (**A**) Western blot analysis of GFP-tagged proteins. Cells expressing GFP-T279A and GFP-T279D were maintained at different G418 concentrations (10, 20 μg/ml). Fifty microgram of the lysate was used for western blot analysis and specific proteins containing GFP were detected using anti-GFP antibody. HM1 lysate was used as a control. (**B**) Amoebic cells with and without indicated constructs were incubated with RBCs for 3 min. The cells were then fixed and immunostained with mice anti-GFP antibody followed by Alexa-488 (green) secondary antibody. F-actin was stained with TRITC-phalloidin (red). The stained cells were viewed using a confocal microscope. Arrow heads indicate phagocytic cups and enrichment of indicated proteins. Star represents attached RBCs. Bar represents 5 μm. (DIC, differential interference contrast). (**C**) RBC uptake assay was performed using indicated cells. The experiments were carried out three times independently in triplicates. ANOVA test was used for statistical comparisons. *p-value ≤ 0.05, **p-value ≤ 0.005, ***p-value ≤ 0.0005. (**D**) Phosphorylated EhAK1 was detected by immunoprecipitation from amoeba lysate over-expressing GFP tagged EhAK1 using anti EhAK1 antibody (1: 1000). Blot 1# Immunoprecipitated material was analyzed by western blotting using phospho-threonine specific antibody (1: 100) and showing two bands, upper 75 kDa GFP-EhAK1 and lower 50 kDa EhAK1. Blot 2# Immunoprecipitated material was analysed by western blotting using anti GFP antibody (1: 1000) and showing only one band at 75 kDa GFP-EhAK1.

The role of autophosphorylation in erythrophagocytosis was studied by over expressing T279A and T279D mutants as GFP tagged molecules in amoebic cells using a constitutive vector system^13^. Over expression of the proteins in the presence of increasing concentration of G418 was confirmed by immunoblot analysis (Fig. 5A). Generally phagocytic cups are initiated within 3 min of incubation of RBCs with *E. histolytica* cells. However, the process was delayed in cells over expressing autophosphorylation-defective mutants (T279A) because no phagocytic cups were observed after 3 min of incubation despite attachment of RBCs to amoebic cells (Fig. 5B). There was also a significant decline (55%) in the level of phagocytosis when one of the mutants (T279A) was over expressed in the presence of G418 as compared with cells carrying only the vector also in presence of G418 (Fig. 5C). The reduction was even greater in cells expressing K85A (68%) (Fig. 5C). Proliferating cells of *E. histolytica* undergo continuous endocytosis; therefore it is likely that the phosphorylated form of EhAK1 will exist in the cells. A phosphospecific antibody (anti p-Thr) was used for detecting phosphorylated form of EhAK1. The specificity of the antibody was determined by probing purified recombinant protein, phosphorylated protein, T279A, and dephosphorylated protein (Fig. S6). The antibody recognized only the phosphorylated form of the protein and did not bind to the non-phosphorylated form of the protein as seen in immunostained western blot (Fig S6). In order to detect phosphorylated status of EhAK1 in amoebic cells over-expressing GFP-tagged EhAK1, EhAK1 was immunoprecipitated from amoebic cell lysate using anti EhAK1 antibody. When immunoprecipitated material was stained with anti P-thr antibody in a western blot, two bands were observed (upper band 75 kDa, GFP-EhAK1 and lower band 50 kDa EhAK1 (Fig. 5D). However, when anti GFP antibody was used for staining only the upper band corresponding to GFP-EhAK1 was seen (blot 2, showing only upper band , GFP-EhAK1) (Fig. 5D), suggesting that proliferating *E. histolytica* cells contain phosphorylated form of EhAK1.

## Discussions

The vast majority of eukaryotic protein kinases have similar catalytic domain structure consisting of twelve conserved sub domains^14^. However, there are eukaryotic protein kinases that display completely different organization and structure. In this report we describe an atypical kinase from protist parasite *E. histolytica*. EhAK1 is involved in the regulation of erythrophagocytosis in *E. histolytica*^8^. The catalytic domain of EhAK1 does not display any similarity with the catalytic domains of the conventional eukaryotic protein kinases. It is, however, strikingly similar to the catalytic domain of myosin heavy chain kinase A (MHCK A) from *Dictyostelium* and has all conserved motifs characteristics of alpha kinases.

Specificity of many kinases, in terms of the site of autophosphorylation and substrate phosphorylation, has been shown to vary depending on cation used. For example, the kinase domain of EGFR^15^ phosphorylates ser or tyr residue depending on the presence of either Mg^2+^ or Mn^2+^ respectively^16^. EhAK1 can also use different cations in both auto- and substrate phosphorylation reactions. Therefore, it is likely that substrate specificity of EhAK1 may also be regulated by divalent cations *in vivo* though we do not have any evidence in support at present. EhAK1 can also use external substrates, such as histone (type III) and MBP. Our results suggest that EhAK1 is a Ser/Thr kinase. However, it is not inhibited by staurosporine confirming atypical nature of this kinase. Low Km (28 ± 2 nM) for ATP suggests that EhAK1 can work optimally at low ATP concentration once the enzyme is activated. This is physiologically relevant as the parasite may encounter unfavourable nutrient conditions where an enzyme working at low ATP concentrations will help the parasite to endocytose nutrients and aid in its survival.

Our results suggest that autophosphorylation has an important role in the regulation of EhAK1 activity and consequently, may be a key step in its function. The three autophosphorylation sites are likely to have different functions, Thr 279 for substrate phosphorylation and Ser 54/Ser 398 for autophosphorylation. Since autophosphorylation can enhance rate of substrate phosphorylation, by phosphorylating a combination of sites it is likely that phosphorylation of different substrates can be manipulated. This is particularly relevant as EhAK1 carries out autophosphorylation by a trans-bimolecular reaction. Since over-expression of inactive kinase led to a dominant negative phenotype it can be inferred that an active kinase is required for phagocytosis. Many protein kinases require phosphorylation for full activity, and this modification can be a critical event in the activation of these enzymes^17^,^18^. Our analysis suggests that the sites present in EhAK1 may not be conserved in others (data not shown here). All these point towards an intricate control of EhAK1 activity through a number of autophosphorylation events and use of different divalent ions. We need to carry out further work to delineate these mechanisms. Since peptide coverage during mass spectrometric analysis was not very high (54%) it is always possible that there may be more phosphorylation sites not detected by us. Our validation experiments using site directed mutagenesis also confirmed phosphorylation sites. Our results also helped us to delineate the function of different phosphorylation sites, for example, Thr 279 was found to be essential for the kinase activity of EhAK1 and also in erythrophagocytosis. Many protein kinases display multiple autophosphorylation sites and there is functional heterogeneity among different phosphorylation sites^9^. For example, phosphorylation sites Thr348 and Ser366 are both involved in eEF2K activity, the mutants showing significant reduction in overall phosphorylation. While Thr 348 mutation reduced autophosphorylation as well as phosphorylation of substrates MH1 peptide and eEF2, different results were seen with Ser 366 mutation. The mutant displayed reduced autophosphorylation and MH1 peptide phosphorylation but not significant effect was seen when eEF2 was used as a substrate^19^,^20^.

EhAK1 does show similarity to alpha kinases, a group of atypical kinases that have propensity to use alpha helical regions as substrates. Some of the, these properties are: 1) no strict requirement of divalent cation, lack of sensitivity to ser/thr kinase inhibitor staurosporine, multiple autophosphorylation sites and presence of some sequence features and conserved residues. However, it also displays a few properties, such as phosphorylation of non helical region of actin not known to be a substrate of alpha kinases and presence of SH3 domain. Therefore it is difficult to conclude at present if EhAK1 is indeed an alpha kinase.

**Figure 6:**
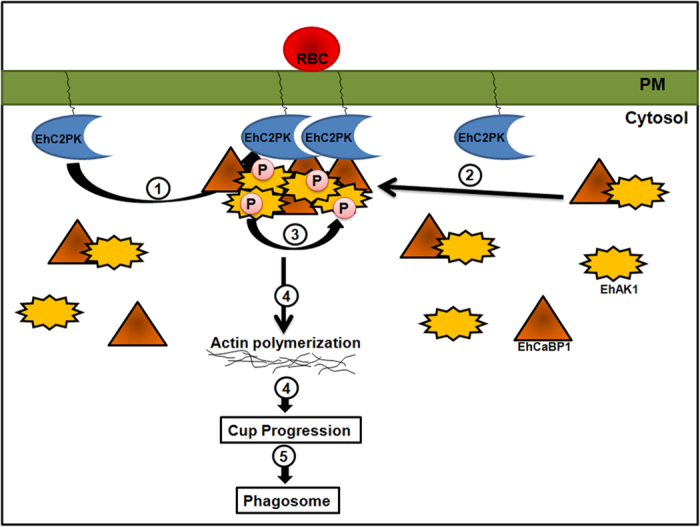
Model depicting phagocytosis of RBC in *E. histolytica*. Model summarises predicted course of events for initiation of phagocytosis in *E. histolytica* based on our experiments. There are other molecules involved in the process, but these are not shown here, for brevity. (**1**) Attachment of RBC to the membrane increases concentration of EhC2PK at the attachment site. (**2**) Recruitment of EhCaBP1 at the site through interaction with EhC2PK. (**2**) Recruitment of EhAK1 at the site through binding with EhCaBP1. (**3**) Activation of EhAK1 via trans-autophosphorylation. (**4**) Actin phosphorylation by EhAK1 and enhanced actin dynamics. (**4**) Progression of cups towards phagosome. (**5**) Phagosome formation.

In conclusion we have biochemically and functionally characterized EhAK1, an atypical protein kinase with some similarity to alpha kinases. The proposed model that shows the suggested involvement of EhAK1 in amoebic phagocytosis is shown in Fig. 6. The pathway is initiated by accumulation of EhC2PK after binding of RBC and recruitment of EhCABP1. EhAK1 is recruited to the phagocytic cups through interaction with EhCaBP1 and activated by autophosphorylation. Phosphorylation of localized G-actin leads to increased rate of polymerization of actin at the tip of the expanding cup, eventually engulfing the particle. This is one of the first protein kinases of *E. histolytica* that have been characterized in detail. This will help in elucidating molecular mechanisms of phagocytosis where EhAK1 is found to participate.

## Materials and Methods

### Kinase Assay

Autophosphorylation of EhAK1 was measured as the amount of radioactivity incorporated i.e. (γ-^32^P-ATP) into the band which co-migrated at with purified recombinant protein. We have already seen that the phosphorylated and non phosphorylated forms of EhAK1 co-migrate in SDS-PAGE. The standard reaction mixture (40 μl final volume) contained 0.5 mM MgCl_2_, 30 mM HEPES (pH 7.5), protease inhibitor, phosphatase inhibitor cocktail and pure kinase (2 μg). Reactions were initiated by the addition of (γ-^32^P-ATP) (6000 Ci/mmol) to a final concentration of 2.5 μM and incubated at 30 °C for 1 h and was stopped by adding SDS sample buffer containing 50 mM EDTA followed by boiling. The samples were than resolved on SDS-PAGE. Phosphorylation was detected by a Phosphor Imager (Fujifilm).

### Immunofluorescence staining

Immunostaining was carried out as described before (Sahoo *et al.*, 2004). Briefly *E. histolytica* cells were harvested via centrifugation and washed with PBS and re-suspended in TYI-33 medium. The cells were then transferred onto acetone-cleaned coverslips placed in a petridish and was allowed to adhere for 10 min at 35.5 °C. The culture medium was removed and the cells were fixed with 3.7% pre-warmed paraformaldehyde for 30 min. After fixation, the cells were permeabilized with 0.1% Triton X-100/PBS for 1 min. The fixed cells were then washed with PBS and quenched for 30 min in PBS containing 50 mM NH_4_Cl. The coverslips were blocked with 1% BSA/PBS for 30 min, followed by incubation with primary antibody at 37 °C for 1 h. The coverslips were washed thrice with PBS followed by 1% BSA/PBS before incubation with secondary antibody for 30 min at 37 °C. Antibody dilutions used were: anti-EhAK1 at 1:100, anti-GFP at 1:200, anti-rabbit Alexa 488 and 556, anti-mice Alexa556 (Molecular Probes) at 1:300, TRITC-Phalloidin at 1:250. The preparations were further washed with PBS and mounted on a glass slide using DABCO (1,4-diazbicyclo (2,2,2) octane (Sigma) 2.5% in 80% glycerol). The edges of the coverslips were sealed with nail-paint to avoid drying. Confocal images were visualized using an Olympus Fluoview FV1000 laser scanning microscope. The raw images were processed using FV10-ASW 1.7 viewer or Image J software.

### Phagocytosis of red blood cells by *E. histolytica* trophozoites

*E. histolytica* trophozoites were harvested in phosphosaline buffer and equal number of amoebic cells i.e. 10^5^ cells were incubated with ten million RBCs which were previously washed with PBS and incomplete TYI-33 for varying times at 37 °C in 0.5 ml of culture medium. The amoebae and erythrocytes were pelleted and non-engulfed RBCs were lysed with cold distilled water and centrifuged at 1000 g for 2 min. This step was repeated twice, followed by resuspending the pellet in 1 ml formic acid to burst amoebae containing engulfed RBCs. The optical density of the samples was determined by spectrophotometry at 400 nm using formic acid as the blank.

### Mass Spectrometry

The purified SDS-PAGE protein band was subjected to in-gel trypsin digestion. Briefly, the excised gel was sliced to small pieces, transferred to a sterile siliconized Eppendorf and destained by repeated washing with 50 mM NH_4_HCO_3_ and 50% acetonitrile. For the reduction, 75 μl of 10 mM stock dithiothreitol was added and incubated for at 55 °C for 30 min. The reduction solution was removed and 50 μl of 50 mM iodoacetamide (IAM) was added and the mixture was incubated at room temperature, in the dark, for 40 min. Enzymatic digestion was carried out by incubating the reaction mixture with trypsin (40 ng/μl of 25 mM NH_4_HCO_3_) and incubation was carried out overnight at 37 °C. After digestion, peptide mixtures were acidified to pH 2.8 with formic acid and desalted with minispin C18 columns (Nestgrp, USA). Samples were dried under vacuum and resolubilized in 0.1% formic acid and 2% acetonitrile before mass spectrometric analysis.

The tryptic peptide samples were separated by reverse-phase chromatography for each via Thermo Scientific Proxeon nano LC using a C18 picofrit analytical column (360 μm OD, 75 μm ID, 10 μm tip, Magic C18 resin, 5 μm size, Newobjective, USA). The HPLC was coupled to an LTQ-Orbitrap Velos mass spectrometer (Thermo Fisher Scientific). Peptides were loaded onto the column with Buffer A (2% acetonitrile, 0.1% formic acid) and eluted with 60 min linear gradient from 2 to 40% buffer B (80% acetonitrile, 0.1% formic acid) followed by 10 min linear gradient from 40% to 80% buffer B and finally 20 min linear gradient from 80% to 90% buffer B. The mass spectra were acquired in the LTQ Orbitrap Velos utilizing Data-Dependent Decision Tree (DDDT) method (Thermo scientific Application Note: 30179) with full MS scan (RP 60000) followed by 20 data-dependent MS/MS scans with detection of the fragment ions in the ITMS mode along with supplemental activation. Target values were 1 × 106 for full FT-MS scans and 3 × 104 for IT-MS MSn scans.

Data analysis was performed using Thermo Proteome Discoverer software suite (1.3.0.339 DBVersion: 78). For the search engine SEQUEST, the peptide precursor mass tolerance was set to 10 ppm, and fragment ion mass tolerance was set to 0.8 Da. Carbamidomethylation on cysteine residues was used as fixed modification, and oxidation of methionine, N-terminal acetylation along with phosphorylation of serine, threonine, and tyrosine was used as variable modifications. Spectra were queried against the *E. histolytica* UniProt database also containing decoy database using a target false discovery rate of 1% for strict and 5% for relaxed conditions. phosphoRS node was used for automated and confident localization of phosphorylation sites within validated peptide sequences.

The mass spectrometry proteomics data have been deposited to the open access library of the ProteomeXchange Consortium (http://www.proteomexchange.org) via the PRIDE partner repository with the dataset identifier PXD002691

### Western blotting

For immunodetection, samples were separated on 10–12% SDS–PAGE as required. The gel was then transferred on to a polyvinylidine fluoride membrane (PVDF) via semi dry method and was further processed using standard methods. The antigens were detected with polyclonal antibodies raised in rabbit or mice as indicated (Anti-EhCaBP1 and EhAK1; 1:1000, Anti-GFP; 1:3000) anti-pThreonine (1:100 custom made from Abmart, china) followed by secondary anti-rabbit and anti-mice immunoglobulins conjugated to HRPO (1:10,000, Sigma). ECL reagents were used for visualization (Millipore). GFP and GST antibodies used were obtained from Molecular probes and Santa Cruz, respectively. The concentration of proteins in a sample was estimated by bicinchoninic acid assay using BSA as a standard.

## Additional Information

**How to cite this article**: Shahid Mansuri, M. *et al.* Autophosphorylation at Thr279 of *Entamoeba histolytica* atypical kinase EhAK1 is required for activity and regulation of erythrophagocytosis. *Sci. Rep.* **6**, 16969; doi: 10.1038/srep16969 (2016).

## Supplementary Material

Supplementary Dataset

Supplementary Information

## References

[b1] Sateriale A. & Huston C. D. A Sequential Model of Host Cell Killing and Phagocytosis by Entamoeba histolytica. J Parasitol Res 2011, 926706 (2011). 21331284 10.1155/2011/926706 PMC3038552

[b2] Okada M. *et al.* Proteomic analysis of phagocytosis in the enteric protozoan parasite Entamoeba histolytica. Eukaryot Cell 4, 827–831 (2005). 15821141 10.1128/EC.4.4.827-831.2005 PMC1087816

[b3] Okada M. & Nozaki T. New insights into molecular mechanisms of phagocytosis in Entamoeba histolytica by proteomic analysis. Arch Med Res 37, 244–252 (2006). 16380325 10.1016/j.arcmed.2005.10.003

[b4] Marion S., Voigt H. & Guillen N. Cellular and biochemical analysis of phagocytosis in Entamoeba histolytica. Arch Med Res 31, S178–180 (2000). 11070273 10.1016/s0188-4409(00)00131-4

[b5] Jain R. *et al.* Calcium-binding protein 1 of Entamoeba histolytica transiently associates with phagocytic cups in a calcium-independent manner. Cell Microbiol 10, 1373–1389 (2008). 18341598 10.1111/j.1462-5822.2008.01134.x

[b6] Somlata Bhattacharya S. & Bhattacharya A. A C2 domain protein kinase initiates phagocytosis in the protozoan parasite Entamoeba histolytica. Nat Commun 2, 230 (2011). 21407196 10.1038/ncomms1199

[b7] Aslam S., Bhattacharya S. & Bhattacharya A. The Calmodulin-like calcium binding protein EhCaBP3 of Entamoeba histolytica regulates phagocytosis and is involved in actin dynamics. PLoS Pathog 8, e1003055 (2012). 23300437 10.1371/journal.ppat.1003055 PMC3531509

[b8] Mansuri M. S., Bhattacharya S. & Bhattacharya A. A Novel Alpha Kinase EhAK1 Phosphorylates Actin and Regulates Phagocytosis in Entamoeba histolytica. PLoS Pathog 10, e1004411 (2014). 25299184 10.1371/journal.ppat.1004411 PMC4192601

[b9] Drennan D. & Ryazanov A. G. Alpha-kinases: analysis of the family and comparison with conventional protein kinases. Prog Biophys Mol Biol 85, 1–32 (2004). 15050379 10.1016/S0079-6107(03)00060-9

[b10] Clark-Lewis I., Sanghera J. S. & Pelech S. L. Definition of a consensus sequence for peptide substrate recognition by p44mpk, the meiosis-activated myelin basic protein kinase. J Biol Chem 266, 15180–15184 (1991). 1907971

[b11] Gonzalez F. A., Raden D. L. & Davis R. J. Identification of substrate recognition determinants for human ERK1 and ERK2 protein kinases. J Biol Chem 266, 22159–22163 (1991). 1939237

[b12] Yao H. & Hersh L. B. Characterization of the binding of the fluorescent ATP analog TNP-ATP to insulysin. Arch Biochem Biophys 451, 175–181 (2006). 16723115 10.1016/j.abb.2006.04.011

[b13] Cormack B. P., Valdivia R. H. & Falkow S. FACS-optimized mutants of the green fluorescent protein (GFP). Gene 173, 33–38 (1996). 8707053 10.1016/0378-1119(95)00685-0

[b14] Hanks S. K. & Hunter T. Protein kinases 6. The eukaryotic protein kinase superfamily: kinase (catalytic) domain structure and classification. FASEB J 9, 576–596 (1995). 7768349

[b15] Wedegaertner P. B. & Gill G. N. Activation of the purified protein tyrosine kinase domain of the epidermal growth factor receptor. J Biol Chem 264, 11346–11353 (1989). 2661557

[b16] Yuan C. J., Huang C. Y. & Graves D. J. Phosphorylase kinase, a metal ion-dependent dual specificity kinase. J Biol Chem 268, 17683–17686 (1993). 8349652

[b17] Nolen B., Taylor S. & Ghosh G. Regulation of protein kinases; controlling activity through activation segment conformation. Mol Cell 15, 661–675 (2004). 15350212 10.1016/j.molcel.2004.08.024

[b18] Olsen J. V. *et al.* Global, *in vivo*, and site-specific phosphorylation dynamics in signaling networks. Cell 127, 635–648 (2006). 17081983 10.1016/j.cell.2006.09.026

[b19] Browne G. J., Finn S. G. & Proud C. G. Stimulation of the AMP-activated protein kinase leads to activation of eukaryotic elongation factor 2 kinase and to its phosphorylation at a novel site, serine 398. J Biol Chem 279, 12220–12231 (2004). 14709557 10.1074/jbc.M309773200

[b20] Pyr Dit Ruys S. *et al.* Identification of autophosphorylation sites in eukaryotic elongation factor-2 kinase. Biochem J 442, 681–692 (2012). 22216903 10.1042/BJ20111530 PMC3286862

