# Supplementary material for: Autophosphorylation at Thr279 of Entamoeba histolytica atypical kinase EhAK1 is required for activity and regulation of erythrophagocytosis
Source: Sci Rep. 2016 Jan 7;6:16969. doi: 10.1038/srep16969 (PMC4703981; doi:10.1038/srep16969)
Supplement: Supplementary Information [file srep16969-s2.pdf]

Supplementary Information:

Autophosphorylation at Thr279 of *Entamoeba histolytica* atypical kinase EhAK1 is required for activity and regulation of erythrophagocytosis

M Shahid Mansuri[1], Mrigya Babuta[1], Mohammad Sabir Ali[1], Ravi Bharadwaj[2], Gagan Deep jhingan, [4] Samudrala Gourinath[1], Sudha Bhattacharya[3] and Alok Bhattacharya[1,]

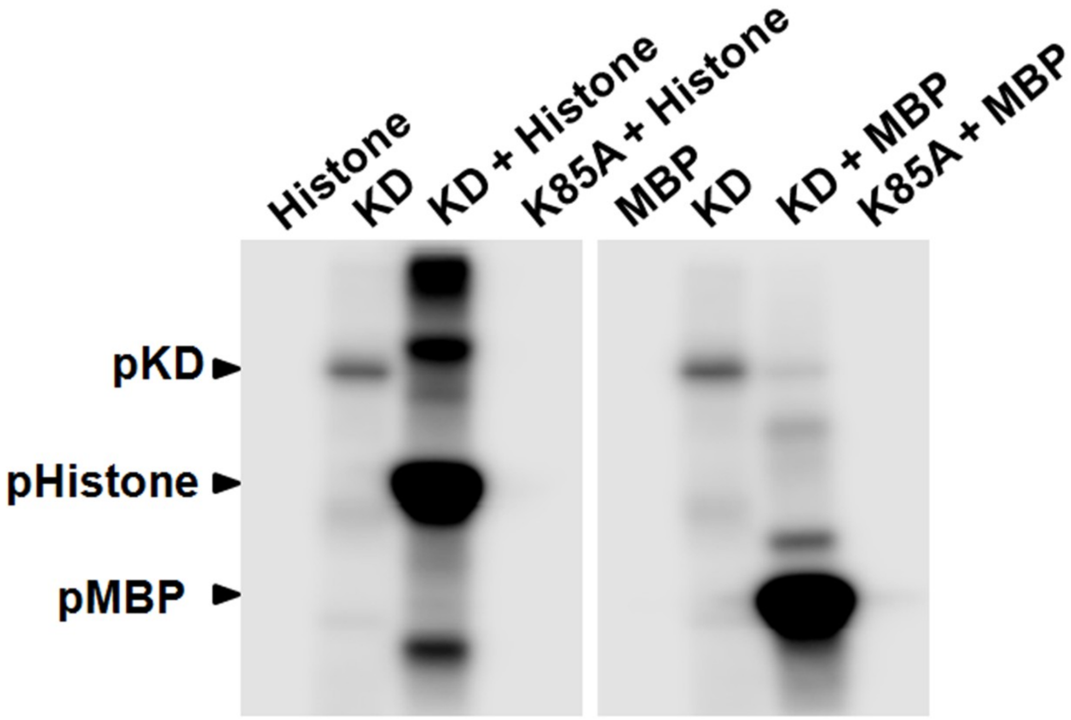

Figure S1. Activity of kinase domain (KD) of EhAK1.
Autophosphorylation and substrate phosphorylation activities of His-tagged kinase domain of EhAK1 were determined. Purified recombinant His-tagged KD or K85A (2μg) was incubated in the presence of γ-32P-ATP, $MgCl_2$ and substrate (2μg) histone type (IIIS) at 30°C for 1h in kinase assay buffer. K85A mutant of EhAK1 exhibits no autophosphorylation and substrate phosphorylation activity. The kinase reaction was stopped by adding Laemmli buffer with 1mM EDTA. The products were analysed on SDS-PAGE and visualized in a phosphorimager.

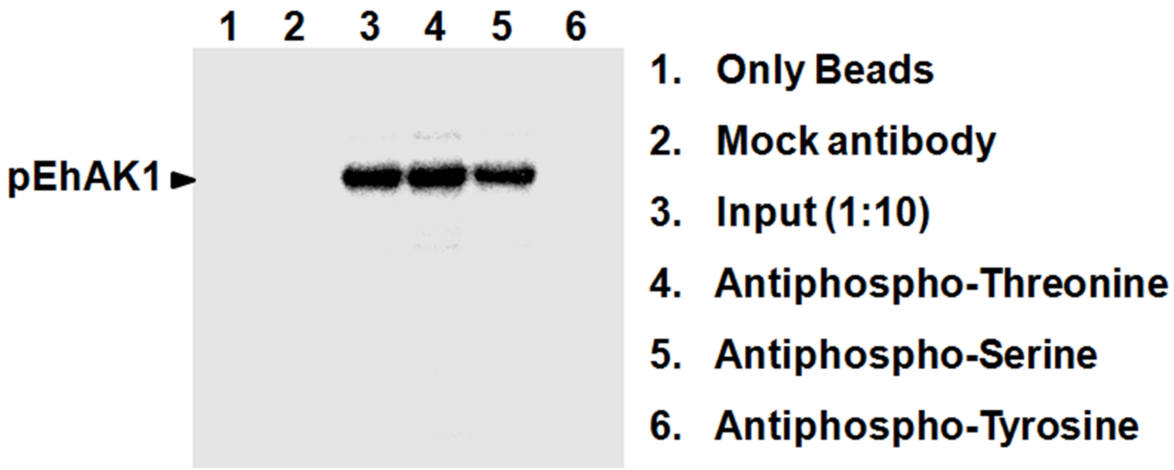

Figure S2. Immunoprecipitation of EhAK1 by Phospho-antibody.
Immunoprecipitation of radio labelled phosphorylated EhAK1 was carried out with respective anti-p-Ser, Thr and Tyr antibodies and analysed by SDS-PAGE. Radiolabeled bands were visualized using a phosphoimager.

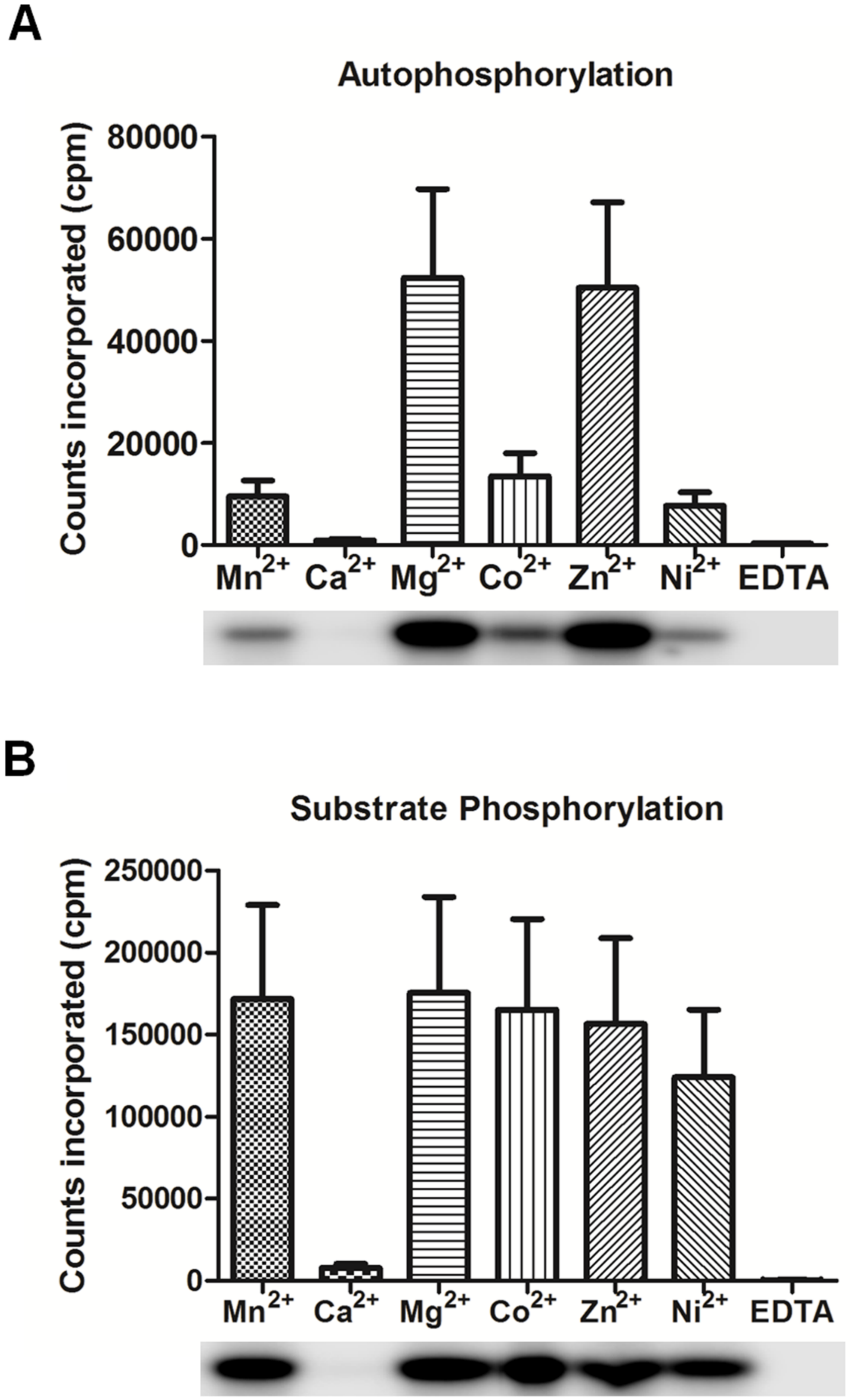

Figure S3. Divalent cation dependence of kinase activity.

(A) and (B) Autophosphorylation reactions were set up as described in panel 2A, in which reaction contained 2µg of His-tagged EhAK1 in the presence of 1mM of indicated ions (A). Substrate phosphorylation was set up with 5µg of histone type (IIIS) in presence of 1mM of indicated divalent cation (B). EDTA used as control. The reactions were stopped by adding

Laemmli buffer containing 1mM EDTA and products were resolved on a SDS-PAGE gel. The gel was stained by coomassie brilliant blue and bands were cut and counts were taken in a scintillation counter.

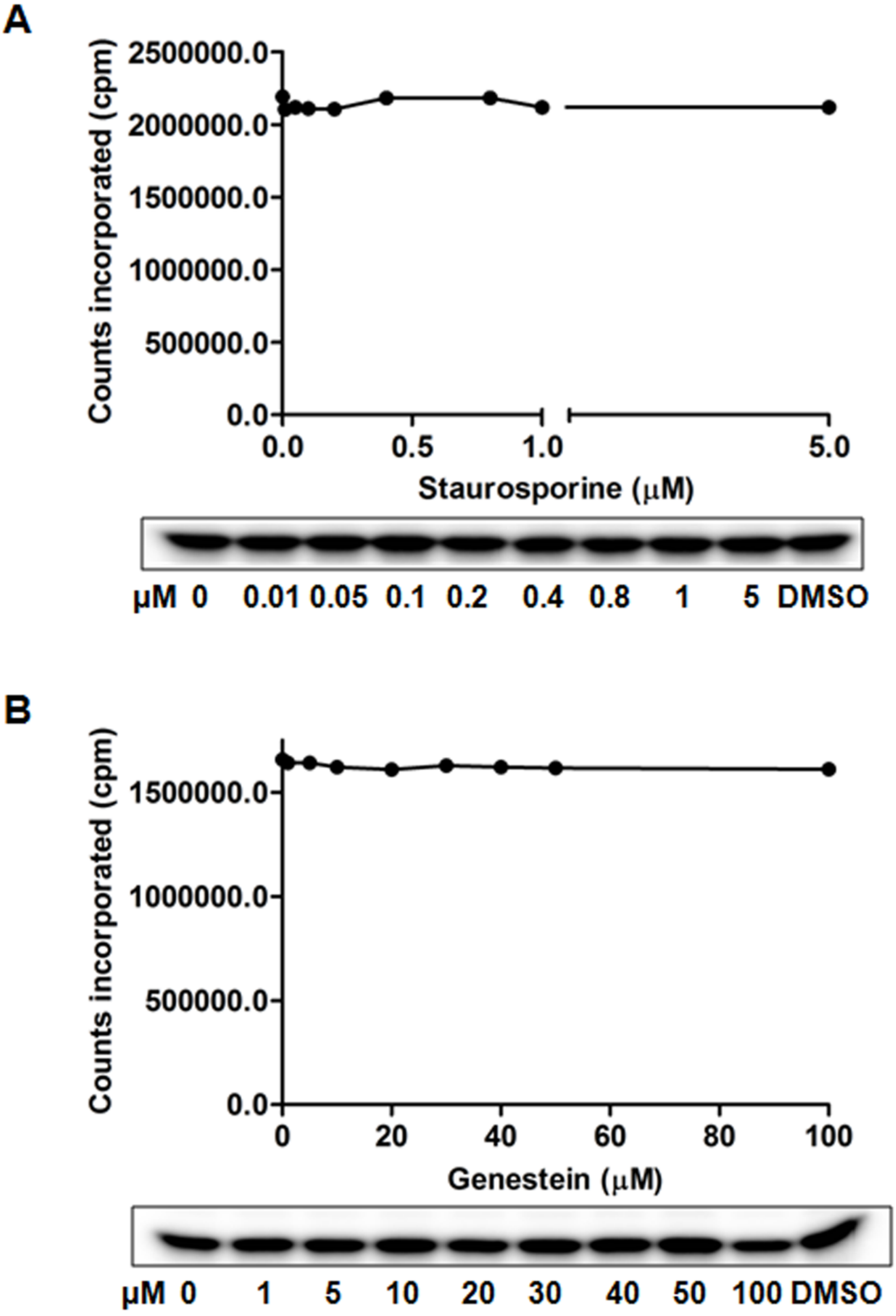

Figure S4. Effect of protein kinase inhibitors on activity of EhAK1.

(A) and **(B)** *In vitro* auto-phosphorylation assay was performed with 2μg purified recombinant His-tagged EhAK1. The protein kinase inhibitors genistein or staurosporine were used as indicated. The reaction was initiated by adding of γ-^{32}P-ATP and was terminated by adding Laemmli buffer with 1mM EDTA. The products were analysed on SDS-PAGE and visualized in a phosphoimager or gel was stained

by coomassie brilliant blue and bands were cut and counts were taken in scintillation counter. DMSO was used as a control.

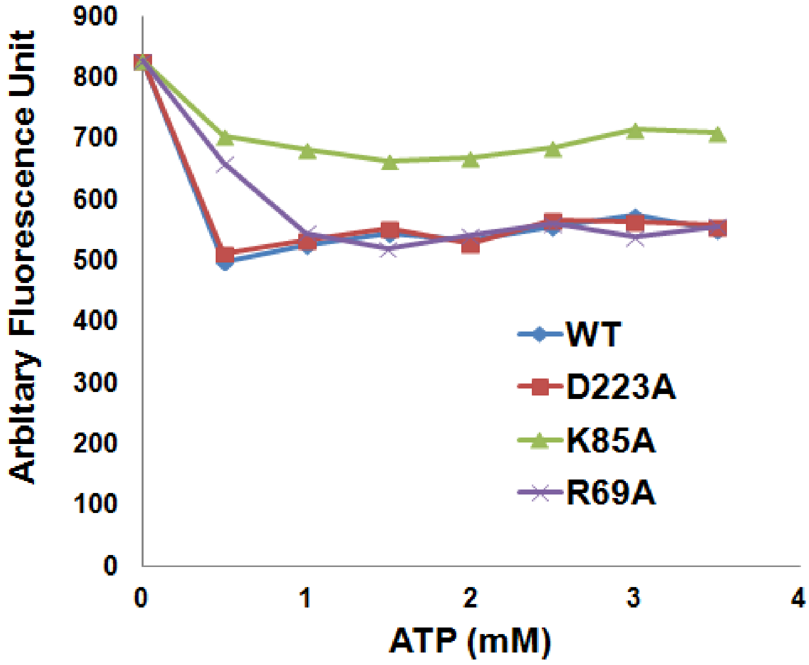

Figure S5. Analysis of ATP binding sites of EhAK1.

Effect of ATP binding on internal fluorescence of EhAK1. Wild type EhAK1 and D223A mutant have ability to bind to ATP while K85A and R69A mutants bind ATP with less efficiency. There is a visible decrease in the fluorescence intensity with increasing ATP concentration. All proteins were taken at concentration of 2.5µM and mixed with 0-3.5mM ATP and 1mM MgCl2 in 10mM HEPES and 100mM NaCl buffer in a reaction volume of 0.7mL. Fluorescence was recorded at 334nm.

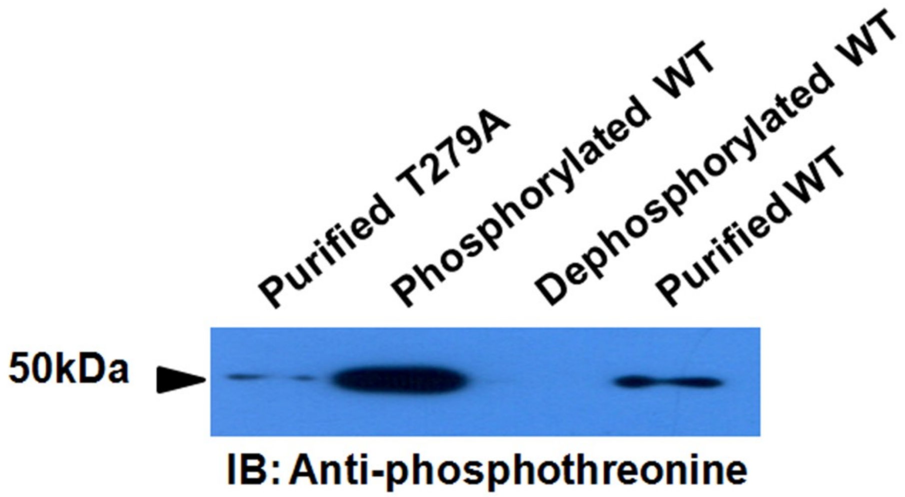

Figure S6. Specificity of anti phospho-threonine antibody.
Purified recombinant EhAK1, phosphorylated EhAK1, dephosphorylated EhAK1 and T279A mutant were separated on a SDS-PAGE and transferred on to a PVDF membrane. These proteins were visualized by immunostaining using phospho-threonine antibody at dilution 1: 100.
